# Nexus of IDO1/Kynurenine Pathway to T-Cell Exhaustion: Hypoxia-Induced Tryptophan Metabolism in Glioblastoma

**DOI:** 10.3390/metabo16030185

**Published:** 2026-03-10

**Authors:** Matthew Abikenari, George Nageeb, Joseph H. Ha, Matthew Adam Sjoholm, Justin Liu, Brandon Bergsneider, Jocelyn Valenzuela, James Poe, Kwang Bog Cho, Rohit Verma, Caren Wu, Vivek Sanker, Ravi Medikonda, Lily H. Kim, John Choi, Matei A. Banu, Michael Lim

**Affiliations:** Department of Neurosurgery, Stanford University School of Medicine, Stanford, CA 94304, USA; mattabi@stanford.edu (M.A.);

**Keywords:** glioblastoma, immunometabolism, tryptophan–kynurenine pathway, IDO1/TDO2, hypoxia (HIF-1α/HIF-2α), GCN2–AHR signaling, tumor microenvironment, metabolic reprogramming, spatial transcriptomics, precision immunotherapy

## Abstract

Glioblastoma (GBM) is a universally fatal cancer for which the standard of care has remained largely unchanged for the last 20 years. Recent work has demonstrated that most therapeutic trials for GBM fail due to complex mechanisms of immunosuppression mediated by both the innate and adaptive immune systems. Various metabolic alterations in the tumor microenvironment help maintain this local and systemic immunosuppression, of which the axis of hypoxia-driven tryptophan degradation has garnered substantial attention over the last decade. This paper synthesizes a much-needed elucidation of the immunometabolic reshaping of glioma, myeloid, endothelial, and lymphoid cell lineages induced by hypoxia. The current paper critically evaluates the role of IDO1/TDO2-mediated breakdown of tryptophan and the consequent accumulation of kynurenine, a metabolite that triggers GCN2- and AHR-mediated CD8+ T-cell exhaustion and supports regulatory T-cell differentiation and expansion. Furthermore, we propose a synthesis of mechanistic evidence that establishes a role for the Trp-GCN2-ATF4-VEGFA axis in hypoxia-induced immunosuppression, supporting that pro-tumoral metabolic dysregulation is directly linked to angiogenesis. In GBM, hypoxia and tryptophan–kynurenine pathway dysregulation operate as an integrated metabolic circuit that drives widespread immunosuppression. These mechanisms can be captured by a metabolic signature shared across nearly every cell type in the GBM microenvironment. Drawing on recent spatial transcriptomic, metabolomic, and pharmacologic studies, we outline how this metabolic axis shapes disease biology and how it can be targeted to restore effective antitumor immunity.

## 1. Introduction

Glioblastoma (GBM) is one of the most aggressive primary malignancies of the central nervous system (CNS), characterized by aggressive local growth, a high degree of metabolic dysregulation, and an immunosuppressive tumor microenvironment (TME) [1,2,3]. Although successive generations of treatments with different mechanisms of action, including cytotoxins, targeted therapies, antiangiogenic therapies, and immunotherapies, have shown promise in preclinical studies and early-phase clinical trials, none of these therapies has been successful in large-scale phase 3 clinical trials [3,4,5]. This translational failure is in large part due to the profoundly immunosuppressive TME present in GBM, which is difficult to recapitulate in preclinical studies. However, there is growing evidence suggesting that metabolic dysregulation, which plays a role in the reprogramming of various metabolic processes among glioma cells, myeloid cells, endothelial cells, and immune cells, is fundamental to the development of therapeutic resistance [6,7].

While glycolytic reprogramming, aberrant prostaglandin signaling, and altered arginine and adenosine metabolism are well-established features of GBM-driven metabolic hijacking, one of the most extensively studied immunometabolic pathways in GBM is the tryptophan–kynurenine (Trp–Kyn) axis [8,9]. This pathway was recently found to represent a major dysregulated metabolic pathway utilized by GBM cells in order to further perpetuate an immunosuppressive milieu. Indoleamine 2,3-dioxygenase 1 and 2 (IDO1/2) and tryptophan 2,3-dioxygenases (TDO2) overexpression in cancer cells, myeloid cells, and endothelial cells in the TME results in local tryptophan degradation. Furthermore, it also leads to general control nonderepressible 2 (GCN2) stress kinase activation, mTORC1 pathway suppression, and T-cell proliferation and attenuation [6,7,10,11]. By simultaneously secreting Kyn, there is an augmented aryl hydrocarbon receptor (AHR)-mediated transcriptional change, conducive to the expansion of regulatory T cells (Treg) and the differentiation of myeloid cells into suppressive subsets, leading to terminal T-cell exhaustion [10,11,12].

While it is evident that the Trp–Kyn axis makes a smaller percentage contribution to total cellular carbon metabolism than do major bioenergetic processes such as glucose metabolism, glycolysis, and OXPHOS, its impact in GBM is disproportionate because it acts as a high-leverage immunoregulatory axis rather than a bioenergetic pathway. In other words, while glucose metabolism, mannose metabolism, and glycosylation pathways, as well as extracellular matrix substrates such as hyaluronic acid metabolism, are primarily involved in meeting the biomass and energy demands of growing tumors, the tryptophan metabolism pathway acts more like a ‘metabolic checkpoint’ that converts inflammatory and nutrient stress signals into kynurenine and tryptophan depletion signals that regulate immune and vascular responses through GCN2-mTOR inhibition and AhR-dependent tolerogenic transcription. Importantly, GBM phenotypic and genotype differences may affect the location and cell types that utilize this pathway in different tumors: IDH wild-type GBM may have patterned IDO1 activity in endothelial and myeloid cell niches that are often cytokine-inducible, while IDH mutant GBM may have a distinct pathway by which R-2-hydroxyglutarate can regulate TDO2 activity in infiltrating macrophages and increase kynurenine production and AhR-dependent immunosuppression. This illustrates how mutational status may have significant implications for localizing and measuring activity of the Trp metabolism pathway in GBM tumors in which glucose metabolism and glycolysis/OXPHOS are dominant processes.

Recent studies further demonstrate that the Trp-GCN2 axis not only induces immune dysregulation but also drives angiogenesis, a hallmark of GBM aggressiveness. In addition, IDO1-mediated tryptophan catabolism promotes the stabilization of GCN2-specific transcriptional networks within glioma cells to induce VEGFA gene expression and increase endothelial tube formation [6,13,14,15,16]. The observation that an IDO-1 inhibitor targeting Trp-GCN2 simultaneously suppresses VEGFA-mediated angiogenesis and suppresses immunosuppressive function underscores how metabolic dysregulation in the GBM TME serves a pro-tumoral role through multiple mechanisms, including nutrient stress, vascular remodeling, and immune system deregulation [14,16,17,18].

One of the primary drivers of metabolic dysregulation in the GBM TME is hypoxia. Hypoxia leads to the stabilization of HIF-1α and HIF-2α, modulation of glycolysis, and induction of checkpoint ligands, tumor-associated macrophages (TAMs), and tumor-associated neutrophils (TANs) [19,20] with aggressive features [16,21,22]. Recent spatial transcriptomics suggest that programs of hypoxia and kynurenine pathway (KP) activation do not occur diffusely but rather within specific metabolic/immune niches in which exhausted CD8+ T cells, Tregs, and angiogenic endothelial cells reside [7,22,23,24].

This review integrates mechanistic insights into GBM immunometabolism, highlighting how tryptophan degradation, hypoxia, metabolic stress responses, and myeloid cell reprogramming orchestrate a program of immune suppression. We further illustrate how understanding these mechanisms can guide the development of precision medicine approaches based on metabolic phenotyping and combinatorial therapies by targeting the metabolic vulnerabilities of immunosuppressive cells in GBM.

## 2. Hypoxia-Driven Immunometabolic Remodeling in the GBM Microenvironment

Hypoxia is a hallmark of the GBM TME and is caused by a combination of rapid cellular turnover, disturbed vascular anatomy, and intratumoral hemorrhage and necrosis [25,26,27,28]. These processes create distinct oxygen gradients in the TME, establishing structural changes inside the GBM TME. Pseudopalisading necrosis is a common histopathologic feature of glioblastoma; it occurs at the site of cell migration driven by hypoxic injury secondary to collapsed microvessels [27,29,30].

The stabilization of hypoxia-inducible factors (HIF-1α and HIF-2α) triggers a coordinated shift in the metabolism of GBM cells. Notably, recent metabolic assays have clarified that HIF-1α increases glycolysis rates, boosts glucose transport through GLUT1 and GLUT3, and suppresses mitochondrial oxidative phosphorylation [31,32,33,34]. As a result, ATP biosynthesis increasingly depends on anaerobic metabolism, producing lactate. Lactate acidifies the extracellular environment, which in turn disrupts T-cell receptor-mediated signaling and suppresses cytokine production and proliferation in infiltrating CD8+ effector T cells [31,35,36].

There is also evidence that hypoxia coordinates dramatic shifts in the myeloid lineage. Single-cell transcriptomics and spatial transcriptomics have demonstrated that the perinecrotic hypoxic niche is enriched in tumor-associated macrophages (TAMs) that express TREM2 and SPP1, which show diminished antigen presentation activity, increased matrix remodeling, and a gene expression program consistent with suppressed phagocytosis and metabolic fitness [37,38,39,40]. These TAMs have high expression of pro-angiogenic genes that create an immunosuppressive neighborhood conducive to tumor growth [23,32]. Importantly, these processes are not static; lactate and hypoxic stress continuously recruit monocytes and induce transcriptomic and metabolic dysregulation to generate these suppressive TAMs [34,41,42].

In cellular environments of the necrotic core, hypoxia induces tryptophan metabolism. While HIF-1α can suppress TDO2 expression in glioma cells, under hypoxic conditions, IDO1 expression is induced in antigen-presenting cells and endothelial cells, thus inducing tryptophan catabolism in stromal cells. IDO1-expressing endothelial cells at the blood–tumor interface can generate a tryptophan-poor, kynurenine-rich milieu that shapes transendothelial immune cell extravasation, particularly innate myeloid trafficking, such as neutrophil recruitment, into the GBM microenvironment [23,43,44,45]. There are multiple outcomes of this metabolic shift: GCN2 stress-response activation, T-cell proliferation inhibition, and AHR-mediated transcriptional reprogramming into exhausted and regulatory T-cells. In addition, KP activity is significantly enhanced and patterned around hypoxia [12,46,47,48]. A consolidated overview of the hypoxia–kynurenine axis and its immunometabolic consequences in glioblastoma is presented in Table 1.

More recent mechanistic studies further elucidate the connection between hypoxia-induced tryptophan metabolism. IDO1-mediated tryptophan catabolism activates GCN2 signaling, which promotes VEGFA transcription, and this nutrient loss drives angiogenesis. Specifically, our understanding of VEGFA-mediated angiogenesis is expanded by evidence demonstrating that IDO1-driven tryptophan depletion directly contributes to vascular remodeling. This Trp–GCN2–VEGFA circuitry connects immune metabolism with vascular remodeling [10,14,46,51,52]. IDO1 inhibition leads to the suppression of GCN2 activation, diminishes VEGFA transcript expression, and reduces endothelial tube formation, thus supporting this interpretation. Furthermore, IDO1 overexpression promotes VEGFA expression, microvascular CD34+ microvessel expansion, and tumor growth, while inhibition abrogates these responses. These data suggest that refractoriness to VEGFA-targeting therapies could occur at least partially due to IDO1-mediated metabolic reprogramming rather than VEGFA redundancy alone [14,53,54,55]. These same pathways are now being targeted in emerging GBM trials, where CAR-T, NK-cell, and myeloid-directed products are engineered to resist such metabolically exhausted cell states. Early-phase results show that metabolic and hypoxia-resistant cell therapies outperform unmodified counterparts, mirroring the mechanisms outlined above [56,57,58].

Overall, hypoxia induces a highly organized state of immunometabolic suppression within GBM. When oxygen deprivation reprograms the metabolic environment of cancers, it induces nutrient-limited stress that causes effector T-cell exhaustion and fosters specific immunosuppressive myeloid subsets. Furthermore, hypoxia synchronously activates tryptophan catabolism in endothelial and myeloid cells by inducing IDO1, thereby increasing kynurenine pathway signaling. In this framework, tryptophan depletion activates GCN2 and ATF4, which transcriptionally upregulates VEGFA and promotes angiogenic programs within the tumor microenvironment. Understanding the interplay between hypoxia, the immune system, and metabolic rewiring could form the basis for strategic therapeutic intervention [9,46,49,59]. Key hypoxia-induced transcriptional and metabolic programs relevant to glioblastoma progression and immune evasion are summarized in Table 2.

## 3. Tryptophan Metabolism in Glioblastoma

### 3.1. Canonical Tryptophan (Trp) Degradation Pathways (IDO1, IDO2, and TDO2)

Tryptophan (Trp) catabolism in GBM is dominated by the kynurenine pathway, initiated by three dioxygenase enzymes: indoleamine 2,3-dioxygenase 1 and 2 (IDO1, IDO2) and tryptophan 2,3-dioxygenase (TDO2) [53,65,66]. These enzymes catalyze the first, rate-limiting step of Trp degradation to N-formylkynurenine, which is quickly converted to kynurenine (Kyn) [58]. Under normal physiology, TDO2 is constitutively active in the liver to maintain Trp homeostasis, whereas IDO1/IDO2 have low basal expression in most tissues but can be rapidly induced by inflammatory cytokines, such as IFN-γ [67]. In GBM, this pathway is frequently upregulated. Gliomas overexpress IDO1/2 and TDO2, leading to local Trp depletion and accumulation of Kyn metabolites [58]. Notably, over 90% of GBMs express IDO1. Importantly, high levels of IDO1/2 or TDO2 correlate with significantly shorter survival. Mechanistically, Kyn acts as an endogenous ligand for the aryl hydrocarbon receptor (AhR), and activation of AhR skews T cells and macrophages toward immunosuppressive phenotypes. Kyn–AhR signaling is implicated in both immune evasion and pro-tumor effects. TDO2-driven Kyn promotes glioma cell migration via AhR-dependent upregulation of the water channel AQP4. Thus, by diverting Trp towards the kynurenine pathway, GBM employs IDO1/IDO2/TDO2 to generate Kyn, a potent mediator of tumor growth and immune evasion [58,67,68].

### 3.2. Regulation of IDO1 Expression in the GBM Microenvironment

Hypoxia serves as a major regulator of Trp metabolism. Low oxygen tension paradoxically represses TDO2 expression in GBM cells. A recent study showed that culturing GBM cells under hypoxia or chemically stabilizing HIF-1α caused a reversible, HIF1α-dependent downregulation of TDO2 mRNA and protein [69]. Additionally, tryptophan metabolism in GBM involves crosstalk between tumor cells, multiple immune cell types, and the surrounding stromal population, especially myeloid immune cells and the vasculature pericytes [70,71]. Inflammatory signals produced by immune cells can upregulate IDO1/TDO2 in other immune compartments, while tumor metabolites affect immune and endothelial cell function. For example, IFN-γ and other cytokines released by infiltrating T cells drive IDO1 expression in both tumor cells and immune cells [60,72].

Hypoxia does not uniformly regulate tryptophan catabolism in the glioblastoma microenvironment in the same direction or to the same extent. Instead, hypoxia has cell-type-specific effects that functionally redirect Trp-catabolizing activity between the tumor and the stroma. For glioma cells, HIF-1α accumulation may repress TDO2 expression, an O_2_-dependent transcriptional response that is characteristic of the malignant cell phenotype. This does not imply that the Trp-Kyn pathway is globally suppressed, however, because the same hypoxic environment is also enriched for signals that drive the activation of the IFN-γ cytokine network, which, in turn, is known to regulate IDO1 expression in the antigen-presenting cell type, as well as specialized endothelial cell types located at the blood–tumor interface. Hypoxia, suppressing TDO2 activity, and inflammatory cytokines regulating IDO1 expression, therefore, are not contradictory claims that describe the same phenomenon, but rather two different processes that occur concurrently, albeit in different cell types, within the glioma microenvironment [14,55,69,72].

Functionally, the result is an increased rate of immunosuppressive Trp catabolism, regardless of the reduced TDO2 activity of the glioma cell type. The consequence of this shift in Trp-catabolizing activity is the creation of highly localized, yet highly potent, tryptophan-depleted, kynurenine-rich environments that, because they occur at the interfaces between the GBM stroma and the infiltrating immune cell type, are highly effective at imposing the stress response (GCN2-mTOR inhibition) as well as kynurenine-AhR-dependent tolerogenic reprogramming of the T-cell phenotype. This is regardless of the reduced TDO2 activity that is characteristic of the hypoxic environment. Hypoxia, therefore, does not separate the location of Trp catabolism (less TDO2 activity in the hypoxic environment) from the overall result (tryptophan depletion, kynurenine-mediated stress, and tolerogenic reprogramming), while preserving, or intensifying, the immunosuppressive phenotype that is the central theme of this review as the critical component of the glioblastoma immune environment [31,32,60,66].

Lastly, endothelial expression of IDO1 at the blood–tumor border is not merely a correlation but may serve as an active filter within the immune microenvironment, creating a localized environment that is low in tryptophan and high in kynurenine at the site where lymphocytes attempt to extravasate into the tumor.

Furthermore, single-cell RNA sequencing of human GBM revealed that IDO1 is predominantly expressed by tumor-associated ECs, concentrated in a subset that co-expresses the chemokine CXCL11 and downstream JAK/STAT pathway genes. Tumor cells often demonstrate minimal IDO1 expression unless stimulated by canonical pro-inflammatory cytokines, whereas the IDO1+ endothelial cluster creates a localized Trp-depleted, Kyn-rich niche at the blood–tumor interface [73]. This endothelial IDO1 activity in GBM can contribute to an immune-privileged microenvironment by hindering T-cell infiltration and function [63].

Myeloid-derived intratumoral cells, including resident microglia, monocyte-derived macrophages, and myeloid-derived suppressor cells (MDSCs), both influence and respond to Trp metabolism. These cells can express Trp-catabolizing enzymes and produce immunosuppressive metabolites such as lactate and adenosine that act on neighboring cells [67]. An illustrative example of tumor–myeloid metabolic crosstalk occurs in IDH-mutant gliomas. Tumor cells secrete the oncometabolite R-2-hydroxyglutarate (R-2-HG), which reprograms infiltrating macrophages by potently enhancing their TDO2 enzymatic activity. In turn, macrophages ramp up kynurenine production from Trp, thereby activating AhR in the tumor microenvironment. This reprogramming induces immunosuppression by skewing macrophages toward a suppressive (M2-like) state that supports Tregs and dampens CD8^+^ T-cell activity, reinforcing a kynurenine-rich niche.

Notably, TDO2-deficient macrophages are resistant to the effects of R-2-HG on T-cell exhaustion [67]. Together, these interactions support the impact of cooperative metabolism in GBM: tumor cells, immune cells, and endothelial cells jointly modulate the Trp–kynurenine pathway to maintain immune tolerance and allow the sustained local and systemic immunosuppression characteristic of GBM. By sharing the enzymatic workload and metabolite exchange, tumor cells ensure that Trp depletion and kynurenine byproducts persist, whether it be via tumor-intrinsic TDO2 activity in normoxic zones, IDO1^+^ macrophages and microglia at the invasive margins, or IDO1^+^ endothelial cells at the tumor’s vascular niche. This metabolic crosstalk ultimately supports tumor immune evasion, highlighting multiple cell types in GBM as potential targets for therapies that interrupt tryptophan metabolism. Table 3 describes major alterations within the tryptophan–kynurenine pathway and their downstream immunologic effects in glioblastoma.

## 4. Kynurenine Pathway and T-Cell Dysfunction

### 4.1. Mechanisms of T-Cell Exhaustion

GBM is characterized by its immunosuppressive tumor microenvironment, which drives effector T-cell dysfunction and eventual exhaustion [77,87,88]. Tumor-infiltrating lymphocytes (TILs) in GBM commonly express multiple inhibitory immune checkpoints, such as PD-1, TIM-3, and LAG-3, which are consistent with an exhausted T-cell phenotype. Co-expression of these receptors on CD8+ T cells has been associated with impaired effector function and proliferation, despite antigen presence [88]. In parallel, GBM imposes metabolic restrictions that starve T cells of energy. One such example is the upregulation by glioma and myeloid cells of IDO1, with rapid Trp depletion. Trp is essential for T-cell growth and function [61]. Furthermore, IDO1-driven tryptophan depletion activates the stress-response kinase GCN2 in T cells, inhibiting mTOR signaling and causing cell cycle arrest and anergy in effector T cells [62,64].

### 4.2. Kynurenine as an AhR Agonist

Opitz et al. first identified Kyn as an endogenous AhR ligand in gliomas, linking tryptophan metabolism to a gene-expressing program that promotes immune tolerance [6]. Kyn enters T cells to bind AhR, triggering transcriptional programs that skew T cells toward a regulatory and exhausted state [78]. AhR activation in CD4+ T cells induces the transcription factor FoxP3, biasing these cells to differentiate into Tregs at the expense of effector phenotypes [12]. In CD8+ T cells, Kyn-AhR signaling upregulates inhibitory receptors such as PD-1 while dampening IL-2 production, thereby reinforcing exhaustion [47,89]. This molecular reprogramming is a key mechanism contributing to T-cell exhaustion, blunting antitumor T-cell activity in GBM.

### 4.3. Evidence of Kynurenine-Mediated Expansion of Tregs and MDSCs

**Figure 1:**
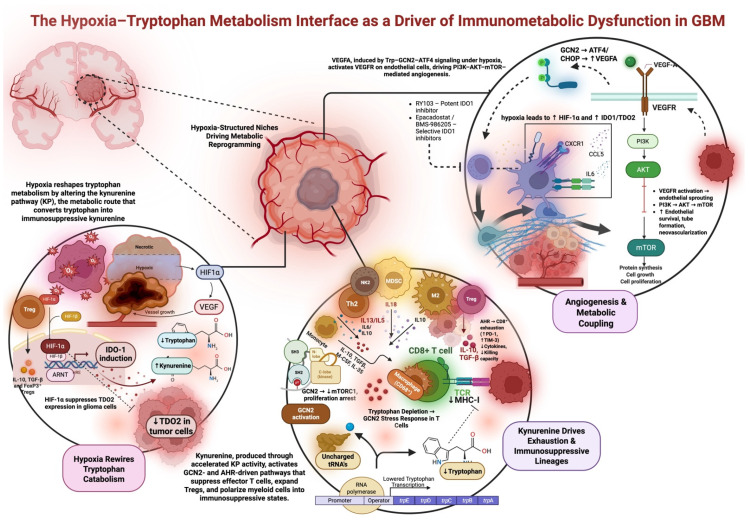
Hypoxia–tryptophan metabolic coupling drives immunosuppression and angiogenesis in GBM. Hypoxia-driven reprogramming of tryptophan metabolism leads to immune suppression and angiogenic remodeling in glioblastoma. HIF-1α stabilization increases IDO1 expression and activity, leading to accelerated flux through the kynurenine pathway, tryptophan depletion, and activation of GCN2/ATF4 stress-response signaling in effector T cells and myeloid cells. This stress response inhibits cytotoxic function and mediates the induction of exhausted T cells as well as the differentiation of immunosuppressive myeloid cells. Furthermore, it induces the differentiation of regulatory T cells and myeloid cells via kynurenine signaling. In tumor and endothelial cells, the activation of GCN2 signaling mediates the induction of VEGFA expression and activation of the VEGFR-PI3K-AKT/mTOR signaling pathway, thus promoting endothelial cell survival and neovascular growth. These interactions collectively establish a hypoxic and immunosuppressive niche in GBM. IDO1 inhibitors, including epacadostat, are key metabolic and signaling nodes that are therapeutically actionable within this pathway.

One hallmark of the GBM tumor microenvironment is the abundance of immunosuppressive cells, notably FoxP3+ Tregs and MDSCs [68,90]. The kynurenine pathway actively drives the expansion and recruitment of these cells. In clinical samples, high IDO1 correlates with elevated Treg infiltration and poorer patient outcomes, indicating that tryptophan catabolism is tied to Treg-mediated immunosuppression [61,68]. In preclinical murine tumor models, IDO1 overexpression promotes the expansion of immature myeloid cells, which, under the influence of infiltrating Tregs, differentiate into immunosuppressive MDSCs [50]. AhR signaling induces CCR2 and other chemokine receptors that facilitate myeloid cell trafficking to Kyn-rich tumor zones [74]. Collectively, these observations demonstrate that Kyn signaling expands the two major immunosuppressive cell populations in GBM, ultimately fostering its characteristic microenvironment, which is refractory to effective antitumor T-cell responses. Figure 1 recapitualtes the hypoxia-driven reprogramming of tryptophan metabolism that leads to immune suppression and angiogenic remodeling in glioblastoma.

## 5. Contemporary Therapeutic Implications

Disruption of IDO1 activity has been investigated as a therapeutic strategy for GBM. Among the available IDO1 inhibitors, epacadostat, indoximod, PF-06840003, and BMS-986205 have received particular attention [91]. Indoximod acts as a tryptophan mimic, disrupting downstream immunosuppressive signaling driven by IDO1 activity [92]. It is important to note that indoximod (D-1-methyl-tryptophan) is not a direct enzymatic inhibitor of IDO1; rather, it acts downstream of IDO-mediated tryptophan depletion by functionally mimicking tryptophan to restore mTORC1 signaling in T cells, thereby countering starvation-induced immune suppression. This downstream mTORC1-rescue mechanism is biologically distinct from direct IDO1 enzyme blockade (e.g., epacadostat) and may help explain why clinical responses can diverge between pathway bypass strategies and attempts to fully suppress kynurenine production at the enzymatic source.

In a phase 1 trial (NCT02502708) evaluating safety and preliminary efficacy, indoximod was well-tolerated and produced a partial response in 1 of 16 patients with GBM [93]. A subsequent phase 1/2 trial (NCT02052648) further demonstrated that indoximod remained well-tolerated, including when administered in combination with temozolomide (TMZ) and stereotactic radiation [94]. Ongoing trials continue to broaden its clinical evaluation: a phase 1 study (NCT05106296) is currently recruiting to test indoximod in combination with a Bruton′s tyrosine kinase (BTK) inhibitor plus chemotherapy, while a phase 2 trial (NCT04049669) is enrolling pediatric patients with gliomas, including GBM, to assess its use alongside chemoradiotherapy [75,95].

Epacadostat directly reduces IDO1 enzymatic activity through competitive inhibition [81,92]. In a phase 1/2 trial (NCT02327078), the combination of epacadostat with PD-1 blockade demonstrated a favorable safety profile [82,96]. A phase 2 clinical trial (NCT03532295) is currently evaluating epacadostat with PD-1 inhibition, radiotherapy, and vascular endothelial growth factor (VEGF) blockade for recurrent glioma. Preliminary findings indicate that this multimodal regimen is also well-tolerated [76].

PF-06840003 and BMS-986205 are two additional small-molecule IDO1 inhibitors that have advanced into early-phase clinical evaluation. The safety of PF-06840003 was assessed in the phase 1 trial NCT02764151. Although the study was ultimately terminated by the sponsor, preliminary data suggested a favorable safety profile [97,98]. BMS-986205 is currently under investigation in combination with PD-1 blockade in an active phase 1 trial (NCT04047706), though no preliminary results have been reported at the time of writing [79]. Despite the growing number of IDO1-targeted agents progressing through clinical development, variable efficacy across studies highlights the need for robust biomarkers capable of identifying patients most likely to benefit from IDO1 inhibition.

Preclinically, nanotechnology offers a promising platform for delivering anti-IDO therapies to GBM. PPRX-1701, a biodegradable polymer loaded with 6-bromoindirubin-3′-acetoxime, has been shown to downregulate IDO1 expression in GBM cells. In immunocompetent murine GBM models, treatment with PPRX-1701 significantly extended survival and reshaped the tumor microenvironment, increasing CD8^+^ T-cell infiltration while reducing immunosuppressive myeloid populations [80]. Nanoparticle-based delivery has also been applied to the genetic targeting of IDO1. For example, a multifunctional envelope-type nanodevice (MEND) has been used to deliver IDO1-specific siRNA into dendritic cells, resulting in efficient gene knockdown and reduced IDO1 expression [99]. Although nanotechnology-based approaches for GBM remain in the early stages of development, successful clinical translation will depend on demonstrating safety, scalability, and clear advantages over conventional drug delivery methods. Nonetheless, the encouraging preclinical results provide a strong rationale for continued innovation in nanoscale delivery of anti-IDO therapies.

To better stratify patients and tailor therapies, researchers are identifying biomarkers that reflect the activity of the IDO1 pathway in GBM. A kynurenine/tryptophan (Kyn/Trp) ratio is a blood-based metabolic index calculated from the levels of l-kynurenine and l-tryptophan, and this measurement holds promise as a diagnostic or prognostic tool and for monitoring treatment response. It serves as a surrogate for IDO/TDO enzymatic activity, with higher IDO1/TDO activity converting more Trp to Kyn. In cancer patients, an elevated Kyn/Trp ratio often signifies an immunosuppressive tumor milieu. In GBM specifically, studies have shown significantly higher Kyn/Trp ratios in patients compared to healthy controls [70,85]. Clinically, a high postoperative Kyn/Trp ratio has been associated with shorter overall survival, underscoring its potential prognostic value [100].

Overall, IDO1 inhibition has demonstrated a favorable safety profile across multiple clinical trials, even when administered alongside highly cytotoxic treatments such as temozolomide, radiotherapy, or immune checkpoint blockade. Despite this encouraging tolerability, clinical responses highlight the need to clarify whether IDO1 inhibition can meaningfully synergize with other therapeutic modalities, including immunotherapy, targeted therapies, and nanotechnology-based delivery systems. In a recent phase 2 trial in recurrent glioblastoma patients, the combination of retifanlimab (anti-PD-1) with hypofractionated radiation and bevacizumab showed promising OS rates (OS-9 of 71.4%) with no dose-limiting toxicities, which supported the favorable safety profile of combining immunotherapy with cytotoxic and antiangiogenic treatments. A subsequent cohort with the IDO1 inhibitor epacadostat was added because of the positive safety profile, with exploratory immunological studies still being conducted. These trials reinforce the fact that the safety of IDO1 inhibitors within combination regimens has been confirmed, but it has not been established under which settings IDO1 inhibitor therapy would provide any added benefits [100].

The limited effectiveness of IDO1 inhibition likely stems from more than just patient selection factors: timing and sequencing may be crucial since the Trp and Kyn axis could become embedded in chronic immune dysfunction. Monotherapies might also be bypassed due to TDO2 and AhR redundancy and insufficient suppression of key pathways in the tumor microenvironment. These mechanistic realities highlight the need to test combination therapies, such as IDO1 inhibitors combined with checkpoint blockers, vascular modulation, and sequencing with radiation therapy, specifically designed to address spatial heterogeneity.

Continued investigation, particularly through biomarker-driven patient selection and rational combination strategies, will be essential to determine whether IDO1-targeted agents can translate their strong preclinical rationale into consistent clinical benefit for patients with GBM. A structured overview of therapeutic strategies targeting hypoxia–kynurenine co-activation in glioblastoma is provided in Table 4.

Lastly, a key translational barrier for targeting the IDO1–kynurenine axis in glioblastoma is that even biologically well-rationalized metabolic therapies can fail when delivery and spatial context are not adequately addressed. In contrast to many other cancers, the GBM tumor microenvironment is isolated from the bloodstream by the BBB, as well as a variable blood–tumor barrier, such that systemic delivery of a compound by no means guarantees adequate tumor concentrations, especially in the infiltrative tumor regions of the tumor microenvironment, where immune–vascular interactions and the IDO1+ endothelial cell niches are present. In parallel, the tumor microenvironment of GBM is highly heterogeneous, with the expression of the IDO1/TDO2 enzymes, as well as the activity of the kynurenine pathway, to be differentially distributed among the tumor, endothelial, and myeloid cell compartments, such that there would be areas of the tumor microenvironment that would be immune-evasive, such as the TDO2/AhR redundant pathways, even if the pathways have been adequately inhibited in other areas of the tumor microenvironment.

## 6. Precision Immunometabolism in GBM

A central challenge going forward is to move away from “one-size-fits-all” IDO1 blockade toward precision immunometabolism targeting in GBM. Recent bulk and single-cell datasets show that IDO1, TDO2, and downstream KP enzymes are heterogeneously expressed across glioma subtypes, with KP activity tightly linked to immunosuppressive myeloid states and T-cell dysfunction [40,57]. Emerging clinical data similarly indicate that high IDO1/TDO2 or AhR expression and elevated Kyn/Trp ratios are associated with shorter survival, highlighting them as tractable biomarkers [65,83,91]. Future studies should therefore define immunometabolic “endotypes” that integrate KP gene/protein expression, hypoxia signatures, and systemic Kyn/Trp ratios with functional readouts of T-cell exhaustion and myeloid polarization. Patients with KP-high, AhR-active, hypoxic tumors could be prospectively assigned to combinations of IDO1/TDO/TDO2 inhibitors, AhR antagonists, or metabolic support of effector T cells in conjunction with PD-1/PD-L1 blockade, whereas KP-low tumors might be steered toward alternative immunotherapies.

Realizing such selection will require resolving where and in which cells tryptophan metabolism and hypoxia coordinate to suppress immunity. Multiplexed ion-beam imaging platforms such as MIBI-TOF already map dozens of proteins at subcellular resolution in solid tumors and have revealed highly structured tumor–immune neighborhoods that predict outcome [84,101,102]. Applying MIBI-TOF or related high-plex imaging to GBM with antibody panels targeting IDO1/TDO2, KP enzymes, hypoxia markers (HIF-1α, CA9), Trp transporters, checkpoint ligands, and lineage/function markers will allow direct visualization of “Kyn factories” and their relationship to exhausted CD8^+^ T cells, Tregs, and suppressive TAM/MDSC states. In parallel, single-cell and spatial transcriptomics can quantify KP and hypoxia gene programs in discrete niches, link them to TCR clonotypes, and track how these architectures remodel after radiotherapy, antiangiogenic therapy, or IDO1/AhR-directed agents [67,86,103,104]. These integrated spatial atlases should supply mechanistically grounded biomarkers (e.g., KP-high hypoxic neighborhoods) to carry directly into trial design.

Finally, hypoxia must be treated as a co-driver rather than a mere contextual factor in IDO1 biology. Preclinical work shows that hypoxia stabilizes HIF-1α, upregulates PD-L1, reshapes the myeloid compartment, and can directly increase IDO1 expression in antigen-presenting cells, raising the possibility that radiotherapy or antiangiogenic regimens that exacerbate hypoxia may potentiate KP-mediated immunosuppression [103,105,106]. Trial schemas should therefore test rational sequencing, such as upfront IDO1/AhR blockade or hypoxia-modulating agents, followed by chemoradiation, and explicitly monitor how hypoxia and KP activity co-evolve. In addition, across CNS tumors, a unifying theme is becoming clear: metabolic function, more than isolated genetic mutations, governs immune suppression, vascular remodeling, and proliferative behavior. These parallels suggest that targeting shared metabolic stress pathways between tumor and immune cells may offer a more effective therapeutic strategy than pathway-specific or receptor-specific strategies alone. Integrating these precision-immunometabolism principles, such as spatial mapping and biomarker-anchored trial design, offers a path to convert our expanding mechanistic understanding of hypoxia-induced tryptophan metabolism into durable clinical benefit for patients with GBM. Lastly, advances in large language models and multimodal AI in oncology raise the realistic prospect of integrating spatial omics with digital pathology to predict immunotherapy response and resistance patterns at the patient level, enabling more precise deployment of immunometabolic interventions in CNS tumors [107]. Lastly, hormone receptor pathways, such as estrogen and progesterone signaling, have been known to converge on PI3K/AKT/mTOR signaling, which is a major controller of glucose uptake, lipid synthesis, and anabolic metabolism. Thus, endocrine agents used in the treatment of meningioma could be repurposed in GBM as metabolic pathway modulators to dampen upstream growth factor/hormone-mediated mTOR signaling [108,109,110,111]. Although PIK3CA mutations are not major GBM drivers, PI3K signaling is hyperactivated in GBM via EGFR signaling/PTEN loss. A rationale for translation to GBM therapy is endocrine therapy in combination with PI3K/mTOR pathway agents to target metabolic adaptability rather than a single-agent approach targeting cell death [81,112,113].

## 7. Conclusions

Hypoxia-induced tryptophan metabolism has emerged as a defining immunosuppressive axis in the GBM TME. By stabilizing HIF signaling and redistributing tryptophan catabolism to endothelial and myeloid compartments, hypoxia influences spatially ordered niches in which the function of effector T cells is selectively impaired, suppressive myeloid states are maintained, and VEGFA-dependent angiogenesis is metabolically coupled to immune escape, further aiding glioma growth. These findings demonstrate that GBM evolution is not defined solely by genetic alterations but by the metabolic circuitry governing cellular behavior within the tumor microenvironment. A logical therapeutic future, therefore, relies on strategies combining metabolic and immune-directed interventions, approaches that co-disrupt hypoxia-driven KP activation, reinstate nutrient availability, and recondition the TME toward effective antitumor immunity.

As the field progresses, therapies directed by spatial phenotyping may facilitate increased therapeutic efficacy and improved outcomes for GBM patients. Lastly, this review aims to encourage two testable predictions arising from this model of metabolism: (i) spatially defined KP-high and hypoxic kynurenine niches may be linked to immune exclusion and poor response, which could be used for patient stratification, and (ii) successful therapy will require an initial sequence that suppresses hypoxia-related pathways (IDO1/AhR, with or without hypoxia modulation), followed by immune reinvigoration. This can be tested prospectively by measuring spatial KP markers (IDO1, TDO2, AhR, and Trp ratios) and correlating these with on-treatment PD assessments to evaluate suppression of key pathways in the tumor microenvironment.

## Figures and Tables

**Table 1:** Hypoxia-driven immunometabolic remodeling in glioblastoma (GBM).

Hypoxia-Driven Process	Key Cellular Effects	Molecular Pathways/Outputs	Representative References
**Formation of hypoxic niches and pseudopalisades**	Migration away from collapsed necrotic microvessels; creation of sharply defined oxygen gradients	ECM remodeling; necrosis; perivascular hypoxia	[20,21,22,25]
**HIF-1α/HIF-2α stabilization**	Shift to glycolytic metabolism; suppression of OXPHOS	Increased GLUT1/GLUT3, increased HK2; reduced mitochondrial respiration	[27,29]
**Lactate accumulation and extracellular acidification**	CD8+ T-cell dysfunction; impaired cytokine production	Lactate-mediated TCR suppression; metabolic paralysis	[30,31,32]
**Reprogramming of TAMs toward SPP1^+^/TREM2^+^ states**	Loss of antigen presentation; increased matrix remodeling	↑(increased)ARG1, ↑MMP9, ↑TFPI2, ↑VEGFA	[11,19,33]
**Hypoxia-driven recruitment and differentiation of monocytes**	Continuous TAM replenishment	Lactate leads to M2-like polarization; adenosine induces immunosuppression	[36,37,38]
**Hypoxia–KP metabolic intersection**	Hypoxia-induced IDO1 in APCs/ECs; TDO2 repression in tumor cells	Tryptophan loss; Kyn accumulation; GCN2 activation	[49,50]

**Table 2:** The tryptophan–kynurenine axis in GBM: cellular sources, immune consequences, and regulatory mechanisms.

Component	Primary Cellular Source(s)	Functional Consequence in GBM	Mechanistic Pathways	References
**IDO1**	Endothelial cells; myeloid cells; tumor cells; T cells (IFN-γ-induced)	Tryptophan depletion; immunosuppression	IDO1 → tryptophan catabolism → increased kynurenine → increased AHR activation	[6,11,60]
**IDO2**	Myeloid cells; select tumor clusters	Additional kynurenine generation; immune dysregulation	IDO2 → increased kynurenine	[54,55]
**TDO2**	Tumor-intrinsic; IDH-mutant TAMs	Promotes migration; kynurenine-mediated suppression	TDO2 → increased kynurenine → AHR activation → increased AQP4	[56,57]
**Kynurenine**	Product of IDO1/IDO2/TDO2	Drives T-cell exhaustion, Treg expansion, MDSC recruitment	Kynurenine → AHR → increased FoxP3, increased PD-1 expression	[10,61,62]
**GCN2 activation**	T cells under tryptophan depletion	Cell cycle arrest; T-cell paralysis	Tryptophan depletion → GCN2 → decreased mTORC1 signaling	[42,63]
**AHR activation**	T cells; TAMs	Treg differentiation; CD8+ exhaustion; suppressive TAM polarization	AHR → increased FoxP3; increased CCR2; increased IL-10	[43,64]
**Hypoxia-linked KP regulation**	APCs; endothelial cells	IDO1 induction; TDO2 repression	HIF-1α → decreased TDO2; increased IDO1	[49,50]

**Table 3:** Therapeutic implications of hypoxia–KP co-activation in glioblastoma.

Therapeutic Strategy	Rationale	Representative Agents/Technologies	Evidence in GBM	References
**Direct IDO1 inhibition**	Block tryptophan degradation; reduce kynurenine; restore T-cell activity	Epacadostat; Indoximod; BMS-986205; PF-06840003	Safe in early trials; limited efficacy without biomarker-driven selection	[74,75,76]
**AHR antagonism**	Prevent kynurenine-induced Treg and TAM reprogramming	AHR inhibitors	Reverses suppressive Treg–TAM axis	[64,77]
**Targeting hypoxia**	Hypoxia amplifies IDO1 expression and immunosuppressive states	HIF-1α/HIF-2α inhibitors	Hypoxia increases PD-L1, IDO1, and exhaustion programs	[18,78]
**Nanotechnology-based IDO1 targeting**	Improve CNS delivery; achieve direct IDO1 suppression	PPRX-1701 nanoparticles; MEND siRNA	Increased CD8+ T cells; decreased suppressive myeloid states	[79,80]
**Combination immune–metabolic therapy**	KP and hypoxia cooperate to suppress effector immunity	IDO1 + PD-1 inhibitors; IDO1 + antiangiogenic therapy	Mechanistic synergy in preclinical and early clinical settings	[81,82]
**Spatial biomarker–guided precision therapy**	Hypoxic/KP-high niches predict therapeutic resistance	Spatial transcriptomics; MIBI-TOF	Identifies compartmentalized “kynurenine factories”	[83,84]
**Metabolic endotyping/patient stratification**	KP-high patients have distinct biology and therapeutic vulnerabilities	Kyn/Trp ratio; IDO1/TDO2/AHR expression	High Kyn/Trp predicts poor overall survival; guides trial design	[85,86]

**Table 4:** Ongoing and completed clinical trials targeting the IDO1–kynurenine pathway in glioma.

NCT ID	Investigational Agent(s)	Target/Mechanism	Population & Setting	Phase
**NCT04047706**	Linrodostat (BMS-986205) + nivolumab + RT ± TMZ	IDO1 inhibitor + PD-1 blockade	ND GBM (IDH-wt; MGMT-unmethylated focus in initial reports)	I
**NCT02052648**	Indoximod + TMZ ± Bev ± stereotactic re-irradiation (SRS/SRT)	IDO pathway modulator	Adult Rec HGG/GBM	I/II
**NCT02764151**	PF-06840003 (oral)	Selective IDO1 inhibitor	Rec malignant glioma	I
**NCT03532295**	Retifanlimab (PD-1) + re-RT + Bev ± epacadostat	Adds IDO1 inhibitor (epacadostat) to PD-1 + RT	Rec GBM/gliosarcoma	II
**NCT02327078**	Epacadostat + nivolumab (± chemotherapy in other arms)	IDO1 inhibitor + PD-1 blockade	Included a GBM cohort with OS endpoints	I/II
**NCT02502708**	Indoximod + TMZ or indoximod + palliative RT	IDO pathway modulator	Pediatric brain tumors, including HGG/DIPG (GBM represented)	I
**NCT04049669**	Indoximod-based chemo-immunotherapy ± RT (adaptive)	IDO pathway modulator	Pediatric: progressive HGG/GBM, MB, ependymoma; ND DIPG cohort	II
**NCT05106296**	Ibrutinib + indoximod (salvage)	BTK inhibitor + IDO pathway modulator	Pediatric brain tumors after prior indoximod	I

## Data Availability

No new data were created or analyzed in this study.

## Abbreviations

GBM	Glioblastoma
HIF-1α	Hypoxia-inducible factor-1 alpha
IDO1	Indoleamine 2,3-dioxygenase 1
Kyn	Kynurenine
GCN2	General control nonderepressible 2 kinase (EIF2AK4)
ATF4	Activating transcription factor 4
Treg	Regulatory T cell
VEGFA	Vascular endothelial growth factor A
VEGFR	Vascular endothelial growth factor receptor
PI3K	Phosphoinositide 3-kinase
AKT	Protein kinase B
mTOR	Mechanistic target of rapamycin
